# Impact of levels of parasitemia and antibodies, acute-phase proteins, as well as stays abroad on hematological and biochemical parameters in 342 dogs with acute *Babesia canis* infection

**DOI:** 10.1186/s13071-025-06997-4

**Published:** 2025-08-15

**Authors:** Imke Maretje von Hohnhorst, Andreas Moritz, Clara Marie Eisenecker, Christina Strube, Kezia Eudora Rodjana, Elisabeth Müller, Ingo Schäfer

**Affiliations:** 1 Clinical Pathology and Clinical Laboratory Diagnostics, Department of Veterinary Medicine, Justus-Liebig-University, Giessen, Germany; 2 Small animal clinic – Internal Medicine, Department of Veterinary Medicine, Justus-Liebig-University, Giessen, Germany; 3 Institute for Parasitology, Centre for Infection Medicine, University of Veterinary Medicine Hannover, Hanover, Germany; 4 Laboklin GmbH & Co. KG, Bad Kissingen, Germany

**Keywords:** Canine babesiosis, Autochthonous, Vector-borne disease, Tick-borne disease, Import, Travel

## Abstract

**Background:**

*Babesia canis* infections are of rising importance in Germany. This retrospective study aimed to correlate hematological and biochemical parameters with acute-phase proteins, levels of parasitemia and antibodies, as well as stays abroad in dogs with acute *B. canis* infection.

**Methods:**

Dogs in Germany tested PCR-positive for *B. canis* and negative for *Anaplasma phagocytophilum* from January 2018 to December 2024 were included if data on hematocrit, leukocytes, and platelets were available. Hematological scoring (HES) was performed by addition of points for mild (+ 1), moderate (+ 2), and marked (+ 3) anemia, thrombocytopenia, and leukopenia, as well as for the presence of pancytopenia (+ 3) and leukocytosis (+ 1). Results of biochemical and CRP analysis, *Babesia* antibody determination, and pathogen quantification were included, if available. *P* ≤ 0.05 in Spearman’s rank correlation was considered statistically significant.

**Results:**

342 dogs were included. History of stays abroad was known for 191/342 dogs (55.8%; no stays abroad 113/191 (59.2%), imported 55/191 (28.8%), travel 23/191 (12.0%)). The most common clinicopathologic findings were increased CRP (87.4%), thrombocytopenia (85.1%), anemia (78.7%), hyperbilirubinemia (74.2%), decreased iron levels (51.1%), and leukopenia (49.7%). Dogs without stays abroad showed significantly higher HES (*n* = 113, median: 6), CRP (*n* = 60, median: 116.2 mg/l), and levels of parasitemia (*n* = 92, median: 2916 × 10^3^ parasites/ml), but lower serum antibody levels (*n* = 59, median: 1.5 TE) compared with imported dogs (HES: *n* = 55, median: 2; CRP: *n* = 23, median: 40.0 mg/l; levels of parasitemia: *n* = 29, median: 23 × 10^3^ parasites/ml; antibodies: *n* = 37, median: 60.6 TE) (*P* < 0.001 each). Positive correlations were found between CRP and levels of parasitemia (ρ = 0.444), CRP and HES (ρ = 0.406), as well as levels of parasitemia and HES (ρ = 0.348), while negative correlations were observed between levels of antibodies and parasitemia (ρ = −0.666), as well as antibody levels and HES (ρ = −0.652) (*P* < 0.001 each).

**Conclusions:**

About 60% of dogs with acute *B. canis* infection had no history of stays abroad, thus representing autochthonous infections. Most dogs without stays abroad were immunologically naive, in contrast to most imported dogs showing positive and high antibody levels. Dogs with high antibody levels showed less severe clinicopathological alterations and lower levels of parasitemia in the peripheral blood, explained by protective antibody activity.

**Graphical Abstract:**

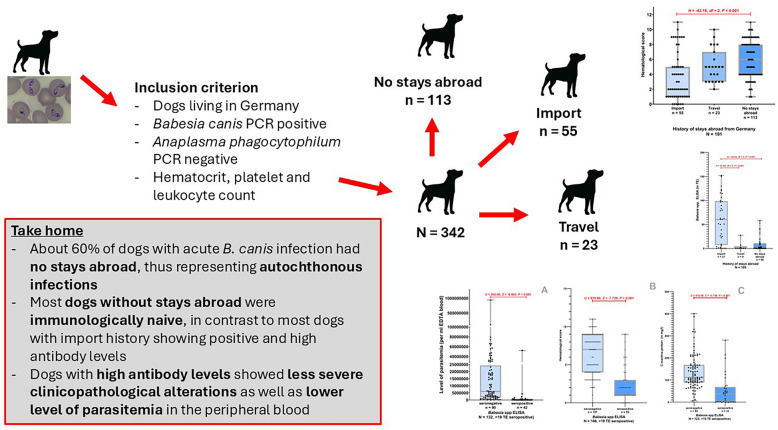

**Supplementary Information:**

The online version contains supplementary material available at 10.1186/s13071-025-06997-4.

## Background

Canine babesiosis in Germany is a tick-borne disease caused by the hemoprotozoan parasite *Babesia canis* [1]. *Dermacentor reticulatus* is the transmitting vector with highest activity levels in spring and autumn [2–4]. Previously, infections with *B. canis* in Germany were mainly linked to dogs with stays abroad in endemic areas [5–7]. Nowadays, acute *B. canis* infections in dogs without stays abroad occur year-round owing to climatic changes and expansion of *D. reticulatus* vector habitats throughout Germany [2, 3, 8, own observation].

Dogs infected with *B. canis* most often show nonspecific clinical signs such as lethargy, fever, and pale to icteric mucous membranes [8, 9, own observation]. Severe intravascular hemolytic anemia associated with pigmenturia was previously considered one of the most remarkable hematological findings [10–12]. Recent studies in Germany demonstrate a predominance of marked thrombocytopenia (94–100%) and mild anemia (82–85%) in *B. canis* infections [8, 13, own observation]. Pancytopenia is also a common finding in 41–45% of dogs [8, 13, own observation].

In biochemical analyses, hyperbilirubinemia (75–90% of cases), azotemia (11–63%), and elevations in ALT (37%), AST (28%), and ALP (3–70%) were observed [8, 13, 14, own observation]. Hypoalbuminemia (58–76%) as a negative acute-phase protein and increased CRP (65–100%) as a positive acute-phase protein are common findings [8, 13, 14, own observation]. In urinalysis, hemoglobinuria is the most remarkable finding, occurring in 26–52% of *B. canis*-infected dogs [8–11, own observation].

For the diagnosis of acute *B. canis* infection, microscopy with detection of erythrocytes infected with *B. canis* and PCR testing are available. Microscopy is a rapid and cost-effective tool, especially in emergency cases. By calculating the percentage of infected cells, low parasitemia < 1% is recognized in peripheral blood in two studies [15, 16]. Higher levels of parasitemia are observed in capillary blood smears, with parasitemia of 12–100% compared with an average of 1.5% in the circulating blood in two other studies [17, 18]. On the basis of microscopical evaluation, levels of parasitemia do not correlate with the severity of clinical manifestations [16], and do not differ between survivors and non-survivors in a more recent study including 15 dogs with natural *B. canis* infections [19]. With PCR testing, high sensitivity and specificity have been reported for detection of *B. canis* DNA from EDTA blood [20, 21]. Species differentiation can be performed in PCR-positive dogs to confirm *B. canis* infection [20]. Pathogen quantification by PCR is additionally available to determine the level of parasitemia via the amount of *Babesia* DNA in the blood [15].

The clinical significance of *Babesia* spp. antibodies determined by IFAT and/or ELISA techniques is unknown. A single positive antibody level can be interpreted as evidence of previous pathogen contact [22–24]. Additionally, potential cross-reactions with other *Babesia* spp. or protozoan parasites such as *Leishmania infantum* must be considered [1, 25]. In acute natural infections, seroconversion takes 3–4 weeks with serologically negative results during this timeframe [1].

The aim of this study was to correlate hematological and biochemical parameters with acute-phase proteins, levels of parasitemia determined by ddPCR, levels of antibodies, and history of stays abroad in dogs with acute *B. canis* infection.

## Methods

Dogs were included in the retrospective study if tested positive for *B. canis* by PCR (forward primer: 5′-AAT ACC CAA TCC TGA CAC AGG G-3′; reverse primer: 5′-TTA AAT ACG AAT GCC CCC AAC-3′, based on Olmeda et al. [26]) and Sanger sequencing, negative for *Anaplasma phagocytophilum* by PCR (TaqMan real-time PCR, target gene: HSP60), and if data for at least hematocrit, leukocyte count, and platelet count were available (Sysmex XN-V analyzer, Sysmex Deutschland GmbH, Norderstedt, Germany) from January 2018 to December 2024. None of the dogs included in the study had been treated with immunosuppressive therapy, antibiotics, or imidocarb dipropionate (as the therapy of choice in acute *B. canis* infections), before blood samples were taken for analysis.

All above-mentioned tests were performed on EDTA blood submitted by veterinarians in Germany to the Laboklin laboratory (Bad Kissingen, Germany). For each dog, only the first blood sample that tested positive for *B. canis* was included. Dogs were excluded if any other *Babesia* species besides *B. canis* was detected by Sanger sequencing.

Each thrombocytopenia below 90 G/l was confirmed microscopically. If available, the results of a biochemical profile—including urea, creatinine, ALT, AST, ALP, GGT, GLDH, bilirubin, total protein, albumin, globulin, CK, triglycerides, iron, DGGR lipase, and CRP (Cobas 8000 analyzer series module c701, Roche Diagnostics, Mannheim, Germany)—as well as *Babesia* spp. antibody levels (Babesia ELISA Dog, Afosa, Blankenfelde-Mahlow, Germany; > 19 TE positive) from serum were additionally included in the evaluation.

If EDTA or purified DNA samples were still available, pathogen quantification was performed retrospectively using a droplet digital PCR (ddPCR, Bio-Rad) targeting the *Bc28.1* gene specific for *B. canis* (forward primer: 5′-GCT ACG TCC GTT GAA GCC-3′ (10 µM), reverse primer: 5′-TCA GCG GAA TAA CGT TCA GC-3′ (10 µM), probe: 5′-FAM-AGC CAG TCG ATC TGC TCC TTT AAG CT-BHQ-3′ (2 µM), based on Kivrane et al. [27]).

For each dog included in the study, a questionnaire was sent to the referring veterinarian to evaluate the medical history, potential stays abroad, clinical signs, therapy, and outcome (Additional file 1: Text S1).

If present, anemia, thrombocytopenia, and leukopenia were classified as mild (0.31–0.43 l/l, 91–149 G/l, 4.0–5.9 G/l), moderate (0.20–0.30 l/l, 40–90 G/l, 2.0–3.9 G/l), or marked (< 0.20 l/l, < 40 G/l, < 2.0 G/l). A hematological score (HES) was calculated by summing the severities of each cytopenia (0 = not present, + 1 = mild, + 2 = moderate, + 3 = marked) as well as the presence of leukocytosis (> 12.0 G/l, 0 = not present, + 1 = present), and pancytopenia (0 = not present, + 3 = present).

For statistical analysis, the software SPSS (version 30.0, IBM) was used and *P* ≤ 0.05 was considered statistically significant. The 95% confidence intervals (CI) were calculated using the Wilson procedure, including continuity correction.

Shapiro–Wilk test was used to assess normal distribution of hematological and biochemical parameters. For comparisons of different dogs, Mann–Whitney *U* test was used for comparison of two groups, and Kruskal–Wallis test including Bonferroni correction for comparison of more than two groups. Spearman-correlation analysis was used to evaluate potential correlations between hematological and biochemical parameters, acute-phase protein (CRP and albumin) concentrations, *Babesia* spp. antibody levels, and levels of *B. canis* parasitemia. Correlations with ρ ≥ 0.5 were considered as strong, ρ = 0.3–0.499 as moderate, and ρ = 0.1–0.299 as mild [28]. The figures were created using GraphPad Prism 10.5.0 (774) for Windows.

## Results

### Study population

A total of 342 dogs with positive *B. canis* PCR were included in this retrospective study. The study population consisted of 176 male (51.5%, 40/176 were castrated) and 154 female (45.0%, 53/154 were spayed) dogs, while the sex of 12 dogs (3.5%) was unknown. The median age of 326/342 dogs (95.3%) was 5 years, ranging from 4 months to 18 years, while the age of the remaining 16 dogs (4.7%) was unknown. The breed was known for 325/342 dogs (95.0%). About one third of the study population were mixed-breed dogs (118/325, 36.3%), whereas the remaining 63.7% (207/325 dogs) were purebred dogs (among others e.g., Labrador Retriever *n* = 22, Golden Retriever *n* = 12, Australian Shepherd *n* = 9, Dachshund *n* = 9, and German Shepherd *n* = 9). A questionnaire on the medical history provided by the referring veterinarian was available for 231/342 dogs (67.5%). Information regarding stays abroad was reported for 191/342 dogs (55.8%). Among them, 113 dogs (59.2%) had no history of stays abroad, 55 dogs (28.8%) were imported, and 23 dogs (12.0%) traveled to other countries. Four imported dogs that traveled after importation were classified in the imported group. The country of origin was known for 52 of the 55 imported dogs (94.5%). Most of these dogs originated from Romania (17/52, 32.7%), followed by Hungary (8/52, 15.4%), Ukraine (4/52, 7.7%), Poland (4/52, 7.7%), Bosnia-Herzegovina (3/52, 5.8%), Greece (3/52, 5.8%), Portugal (2/52, 3.8%), France (2/52, 3.8%), Spain (2/52, 3.8%), and the Netherlands (2/52, 3.8%). One dog each was imported from Belarus, Bulgaria, Italy, Croatia, and Serbia (1.9% each).

### Hematological, biochemical, and CRP analyses

**Fig. 1:**
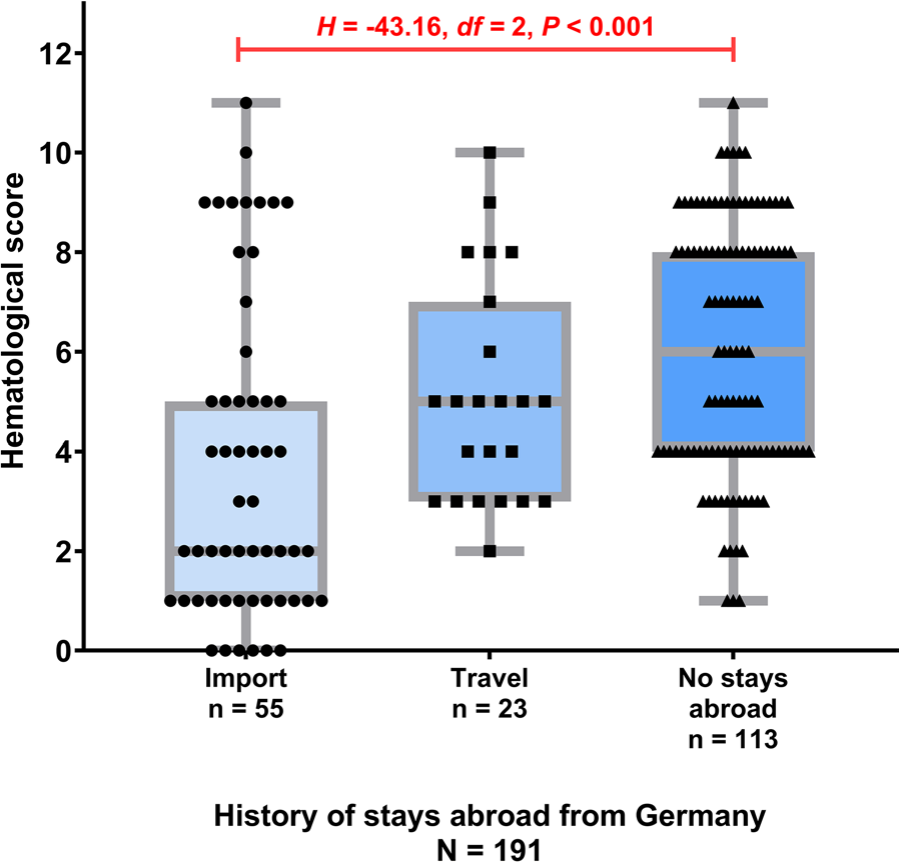
Hematological score (HES, summarizing the severity of each cytopenia: 0 = not present, + 1 = mild, + 2 = moderate, + 3 = marked), presence of leukocytosis (0 = not present, + 1 = present) and pancytopenia (0 = not present, + 3 = present)) in 191 dogs with acute *Babesia canis* infection in Germany and known history regarding stays abroad (Kruskal–Wallis-test including Bonferroni correction *H* = 22.93, *df* = 2, *P* < 0.001)

Hematological results, classified as inclusion criteria, were available for all 342 dogs included in the study (Tab. 1). The most common finding was thrombocytopenia, which was present in 291/342 dogs (85.1%, 95% CI 80.9—88.5%). Thrombocytopenia was most frequently marked (166/342 dogs; 48.5%, 95% CI 43.3—53.8%), less frequently moderate (101/342 dogs; 29.5%, 95% CI 25.0—34.6%), and rarely mild (24/342 dogs; 7.0%, 95% CI 4.8—10.2%). In contrast, anemia (269/342 dogs; 78.7%, 95% CI 74.0—82.7%) was mild in most cases (190/342 dogs; 55.6%, 95% CI 50.3—60.7%), and rarely moderate (67/342 dogs; 19.6%, 95% CI 15.7—24.1%) or marked (12/342 dogs; 3.5%, 95% CI 2.0—6.0%). Leukopenia was observed in 170/342 dogs (49.7%, 95% CI 44.4—55.0%), which was predominantly mild (93/342 dogs; 27.2%, 95% CI 22.7—32.1%) to moderate (71/342 dogs; 20.8%, 95% CI 16.8 —25.4%), while leukocytosis occurred in 45/342 dogs (13.2%, 95% CI 10.0—17.2%). Pancytopenia was diagnosed in 135/342 dogs (39.5%, 95% CI 34.4—44.7%). The HES ranged from 0 – 11 (median: 5), with higher values in dogs without stays abroad/travel to other countries (*n* = 113, range: 1—11, median: 6, SD: 2.5) respectively, compared with imported dogs (*n* = 55, range: 0—11, median: 2, SD: 3.2) (*U* = 2774.50, *Z* = −4.38, *P* < 0.001). No statistically significant impact was observed when comparing traveled dogs (*n* = 23, range: 2 – 10, median: 5, SD: 2.2) to imported dogs (*H* = −26.38, *df* = 2, *P* = 0.158), and dogs without stays abroad/travel to other countries (*H* = -16.77, *df* = 2, *P* = 0.544), but comparing imported dogs to dogs with stays abroad showed significant impact (*H* = −43.16, *df* = 2, *P* < 0.001) (Fig. 1).

**Table 1:** Hematological parameters in 324 dogs with acute *Babesia canis* infection

Parameter	Reference value		Median	Range	Standard deviation	Increased values (%)	Decreased values (%)
Red blood cells		5.5–8.5	5.35	0.83–8.69	1.35	2 (0.6)	195 (60.2)
Hemoglobin (g/l)		150–190	123	19–205	32.2	8 (2.5)	279 (86.1)
Hematocrit (l/l)		0.44–0.52	0.37	0.09–0.61	0.09	13 (4.0)	269 (78.7)
White blood cells (G/l)		6.0–12.0	6.0	1.1–31.5	4.9	45 (13.9)	170 (49.7)
Platelet count (G/l)		150–500	41.0	0.0–777.0	94.6	3 (0.9)	291 (85.1)

Note that pancytopenia occurred in 135/324 dogs (39.5%)

Biochemical results were available for varying numbers of dogs (Tab. 2). The most remarkable findings included hyperbilirubinemia (204/275 dogs; 74.2%, 95% CI 68.7–79.0%), hypoproteinemia (149/291 dogs; 51.2%, 95% CI 45.5–56.9%), azotemia (increased creatinine, 54/250 dogs, 21.6%, 95% CI 16.9–27.1%; increased urea 113/250 dogs, 45.2%, 95% CI 39.1 –51.4%) and slightly elevated liver enzymes (ALT 53/249 dogs, 21.3%, 95% CI 16.7–26.8%; AST 148/235 dogs, 63.0%, 95% CI 56.6–68.9%; ALP 52/244 dogs, 21.3%, 95% CI 16.6–26.9%; GGT 4/151 dogs, 2.6%, 95% CI 1.0–6.6%; GLDH 27/238 dogs, 11.3%, 95% CI 7.9 – 16.0%). Roughly half of the dogs had decreased iron levels (143/280 dogs, 51.1%, 95% CI 45.2–56.9%), and increased DGGR lipase levels were detected in 69/249 dogs (27.7%, 95% CI 22.5–33.6%).

**Table 2:** Biochemical parameters in dogs with acute *Babesia canis* infection

Parameter	Number of dogs	Reference value	Median	Range	Standard deviation	Increased values (%)	Decreased values (%)
Urea (mmol/l)	250	3.3–8.3	7.3	3–82.5	12.2	113 (45.2)	7 (2.8)
Creatinine (μmol/l)	250	< 125	79.5	16–997	111.4	54 (21.6)	–
ALT (U/l)	249	< 88	50.8	10–584	81.2	53 (21.3)	–
AST (U/l)	235	< 51	70.1	8–3269	229.2	148 (63.0)	–
ALP (U/l)	244	< 147	112.5	12–1161	134.8	52 (21.7)	–
GGT (U/l)	151	< 10	1.1	0.1–49.8	5.0	4 (2.6)	–
GLDH (U/l)	238	< 8	3.4	0.4–79.1	7.3	27 (11.3)	–
Bilirubin (μmol/l)	275	< 3.4	6.2	0.2–706.5	50.3	204 (74.2)	–
Total protein (g/l)	291	54–75	53.8	26–92	8.5	3 (1.0)	149 (51.2)
Albumin (g/l)	267	25–44	31.2	16–44.6	4.9	1 (0.4)	27 (10.5)
Globulin (g/l)	257	< 45	22.2	10–67.8	6.4	2 (0.8)	–
CK (U/l)	236	< 200	173.4	27–5214	558.2	107 (31.3)	–
Triglycerides (mmol/l)	207	< 3.9	1.0	0.24–5.63	0.8	4 (1.9)	–
Iron (μmol/l)	280	15–45	14.5	4.2–59.8	11.2	10 (3.6)	143 (51.1)
DGGR lipase (U/l)	249	< 120	61.2	10.3–2524	352.1	69 (27.7)	–
CRP (mg/l)	135	< 15	102	0.2–400	84.5	118 (87.4)	–

*ALT* alanine transaminase, *AST* aspartate transaminase, *ALP* alkaline phosphatase, *GGT* gamma-glutamyl transferase, *GLDH* glutamate dehydrogenase, *CK* creatine kinase, *CRP* C-reactive protein

**Fig. 2:**
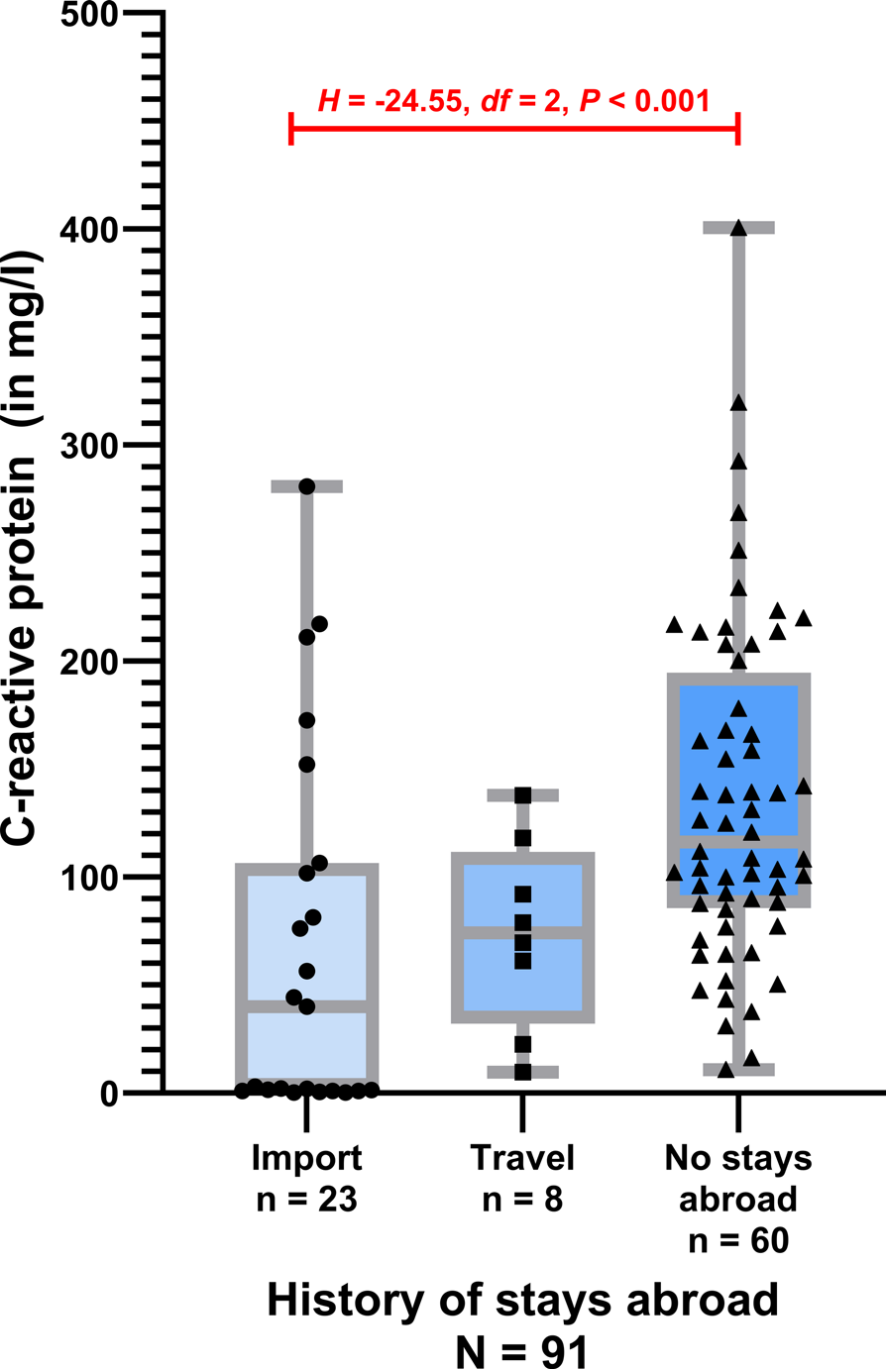
C-reactive protein levels in dogs with acute *Babesia canis* infection comparing imported dogs, traveled dogs, and dogs from Germany without stays abroad (Kruskal–Wallis-test including Bonferroni correction *H* = 16.25, *df* = 2, *P* < 0.001)

Most dogs had elevated CRP values (118/135 dogs; 87.4%, 95% CI 80.8 – 92.0%). The level of CRP elevation differed significantly between dogs with or without stays abroad/travel (Fig. 2). Dogs with no stays abroad/travel to other countries showed higher values (*n* = 60, range: 10.8—400.5 mg/l, median: 116.2 mg/l, SD: 78.1 mg/l) than imported dogs (*n* = 23, range: 0.2–281 mg/l, median: 40.0 mg/l, SD: 85.1 mg/l) (*U* = 451.00, *Z* = −4.01, *P* < 0.001). No statistically significant differences were observed comparing dogs with travel history (*n* = 8, range: 9.6–137.7 mg/l, median: 74.2 mg/l, SD: 43.6 mg/l) to dogs without stays abroad (*H* = −20.23, *df* = 2, *P* = 0.125) as well as to imported dogs (*H* = −4.32, *df* = 2, *P* = 1.000), but statistical significant differences were shown for comparing imported dogs with dogs without stays abroad (*H* = −24.55, *df* = 2, *P* < 0.001) (Fig. 2).

### Antibody levels

**Fig. 3:**
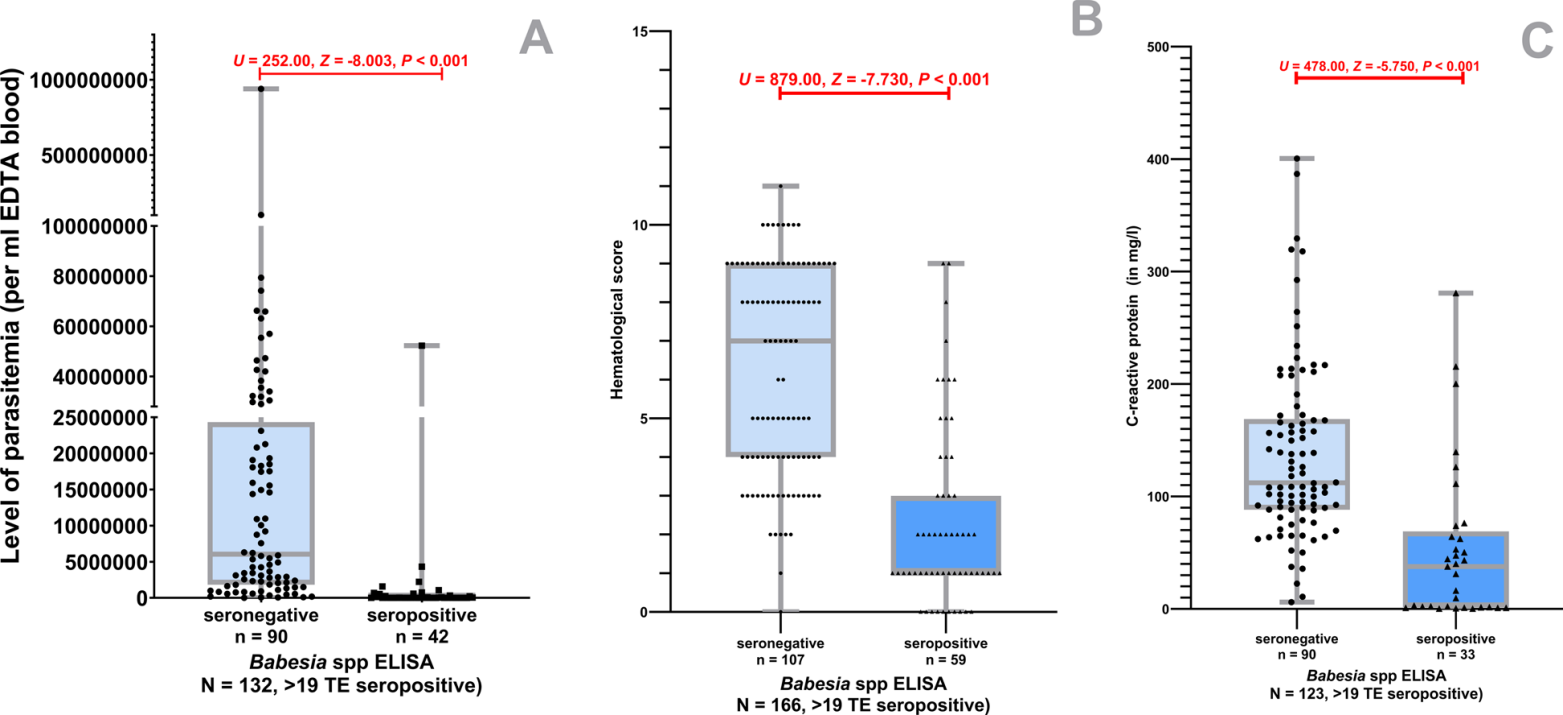
*Babesia* spp. antibody ELISA test results compared with level of parasitemia (**A**, one outlier in negative results not shown with 1,020,000 × 10 ^3^ parasites/ml EDTA blood), the hematological score (**B**), and C-reactive protein (**C**) in dogs with acute *Babesia canis* infection (Mann–Whitney-U-test)

In total, results of *Babesia* spp. antibody testing were available for 166/342 dogs (48.5%, 95% CI 43.3–53.8%). Fifty-nine out of 166 dogs (35.5%, 95% CI 24.2–38.1%) had positive antibody levels > 19 TE. A statistically significant higher CRP concentration (*U* = 478.00, *Z* = −5.75, *P* < 0.001), a higher HES (*U* = 879.00, *Z* = −7.73, *P* < 0.001), and higher levels of parasitemia (*U* = 252.00, *Z* = -8.00, *P* < 0.001) were observed in seronegative dogs compared with seropositive dogs (Fig. 3).

**Fig. 4:**
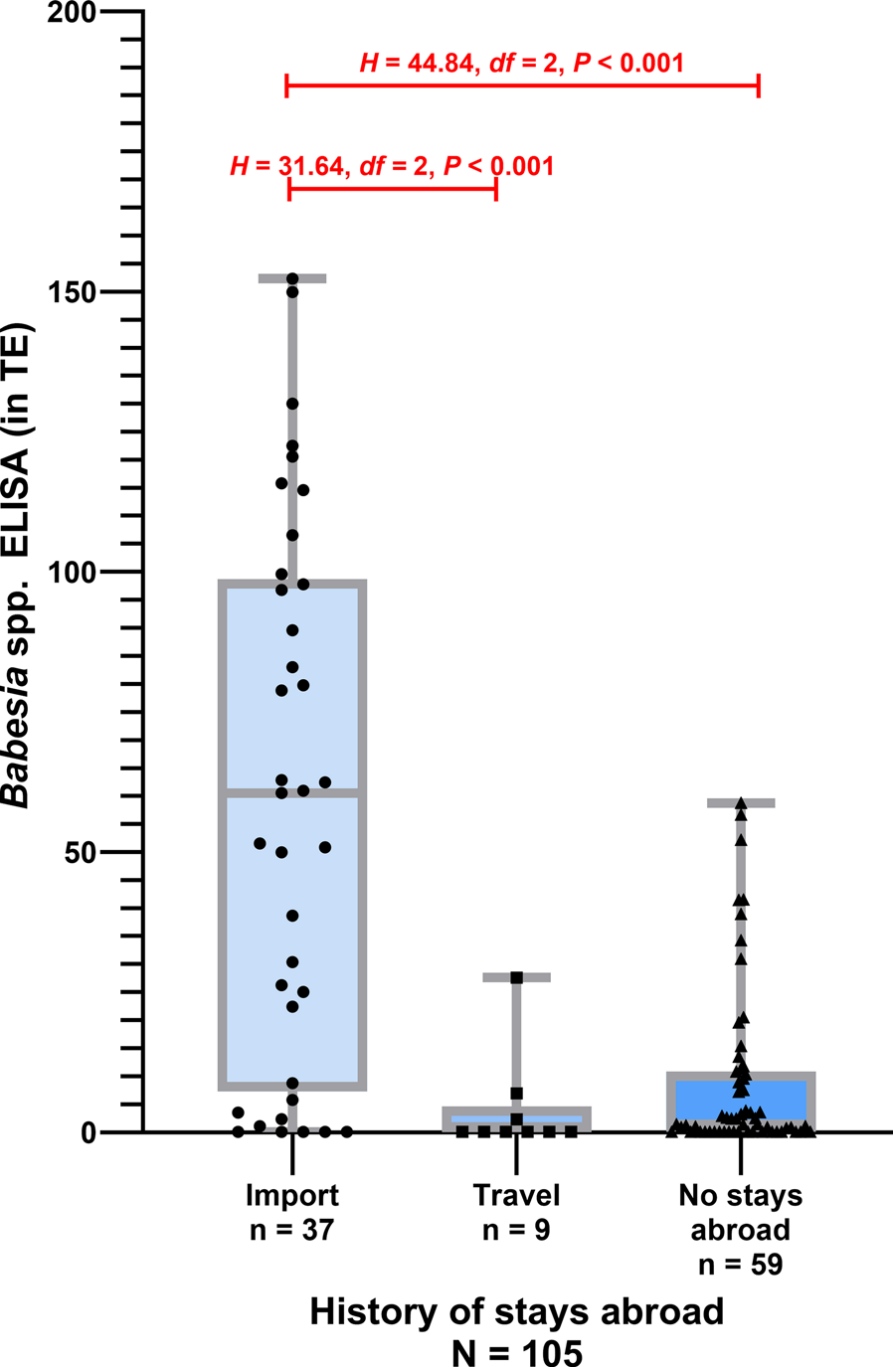
*Babesia canis* antibody levels in dogs with acute *Babesia canis* infection classified according to the history of stays abroad. Antibody levels above the red line (> 19 TE) are considered seropositive (Kruskal–Wallis-test including Bonferroni correction *H* = 31.06, *df* = 2, *P* < 0.001)

Statistically significant differences in antibody levels were observed between imported dogs (*n* = 37, range: 0.1–152.3 TE, median: 60.6 TE, SD: 47.9 TE) and dogs without stays abroad/travel (*n* = 59, range: 0.1–58.8 TE, median: 1.5 TE, SD: 15.3 TE) (*U* = 1448.00,* Z* = 3.87, *P* < 0.001), between imported dogs and traveled dogs (*n* = 9, range: 0.1–27.6 TE, median: 0.1 TE, SD: 9.1 TE) (*H* = 44.84, *df* = 2, *P* < 0.001), and between imported dogs and dogs without stays abroad (*H* = 31.64, *df* = 2, *P* < 0.001). Dogs in Germany without any history of stays abroad/travel were predominantly seronegative (49/59 dogs; 83.1%, 95% CI 71.5—90.5%), while almost three-quarters of dogs with import history tested seropositive (27/37 dogs; 73.0%, 95% CI 57.0—84.6%) (Fig. 4). No statistically significant impact on antibody levels was detected between dogs without stays abroad and traveled dogs (*H* = −13.21, *df* = 2, *P* = 0.659). Of the nine dogs that had traveled abroad, only one dog (11.1%) was seropositive.

### Levels of parasitemia

Pathogen quantification was performed in 222/342 dogs (64.9%, 95% CI 59.7—69.8%) and ranged from 0.07 × 10^3^—1,02 × 10^9^ parasites/ml EDTA blood (median: 1,704 × 10^3^ parasites/ml). In 133/222 dogs (59.9%, 95% CI 53.3—66.1%), history regarding stays abroad was available. Dogs from Germany without stays abroad/travel (*n* = 92; range: 3.8 × 10^3^—1,02 × 10^9^ parasites/ml; median: 2,916 × 10^3^ parasites/ml; SD 106,705 × 10^3^ parasites) showed higher levels of parasitemia than imported dogs (*n* = 29; range: 0.09–74,200 × 10^3^ parasites/ml; median: 23 × 10^3^ parasites/ml; SD 19,638 × 10^3^ parasites) (*U* = 1225.00, Z = −3.221, *P* = 0.001). No significant difference was observed between imported and traveled dogs (*n* = 12; range: 70–31,900 × 10^3^ parasites/ml; median: 1,831 × 10^3^ parasites/ml; SD 11,258 × 10^3^ parasites) (*H* = -17.14, *df* = 2, *P* = 0.585), and between traveled dogs and dogs without stays abroad/travel (*H* = -11.19, *df* = 2, *P* = 1.000).

### Correlation analysis

Parts of the hematological and biochemical parameters showed a normal distribution (*P* > 0.05; erythrocytes, hematocrit, hemoglobin, total protein, albumin, globulin), while all others deviated from normal distribution (*P* ≤ 0.05). Spearman’s correlation analysis was used to correlate the HES, biochemical parameters, acute-phase proteins, levels of parasitemia, and *Babesia* spp. antibody levels (Table 3, Additional file 2).

**Table 3:** Correlation analysis (Spearman-Rho) showing moderate (> 0.3–0.499) to strong (≥ 0.5) correlations of laboratory parameters in dogs with acute *Babesia canis* infection (a = correlation coefficient ρ, b = significance (two-sided), c = n, asterisks mark statistically significant *P*-values < 0.05)

		HES	ALT	ALP	AST	CK	GGT	Bil	Iron	TP	Glob	CRP	AB levels	Ii
HES	a	1.000	0.168^*^	0.343^*^	0.413^*^	0.265^*^	−0.474^*^	0.465^*^	−0.306^*^	−0.357^*^	−0.220^*^	0.406^*^	−0.652^*^	0.348^*^
	b		0.008	< 0.001	< 0.001	< 0.001	< 0.001	< 0.001	< 0.001	< 0.001	< 0.001	< 0.001	< 0.001	< 0.001
	c	342	249	244	235	236	151	275	280	291	257	135	166	222
ALT	a	0.168^*^	1.000	0.538^*^	0.651^*^	0.343^*^	−0.031	0.349^*^	−0.092	−0.051	−0.033	0.149	−0.441^*^	0.347^*^
	b	0.008		< 0.001	< 0.001	< 0.001	0.710	< 0.001	0.160	0.427	0.612	0.100	< 0.001	< 0.001
	c	249	249	243	235	235	151	231	236	246	242	123	147	188
ALP	a	0.343^*^	0.538^*^	1.000	0.561^*^	0.344^*^	−0.064	0.488^*^	−0.048	−0.221^*^	−0.051	0.248^*^	−0.429^*^	0.340^*^
	b	< 0.001	< 0.001		< 0.001	< 0.001	0.432	< 0.001	0.462	0.001	0.430	0.006	< 0.001	< 0.001
	c	244	243	244	234	236	151	232	236	244	242	123	146	184
AST	a	0.413^*^	0.651^*^	0.561^*^	1.000	0.699^*^	−0.420^*^	0.746^*^	−0.213^*^	−0.269^*^	−0.165^*^	0.441^*^	−0.665^*^	0.587^*^
	b	< 0.001	< 0.001	< 0.001		< 0.001	< 0.001	< 0.001	0.001	< 0.001	0.012	< 0.001	< 0.001	< 0.001
	c	235	235	234	235	234	151	229	234	235	233	116	139	178
CK	a	0.265^*^	0.343^*^	0.344^*^	0.699^*^	1.000	−0.243^*^	0.500^*^	0.040	−0.263^*^	−0.133^*^	0.248^*^	−0.450^*^	0.322^*^
	b	< 0.001	< 0.001	< 0.001	< 0.001		0.003	< 0.001	0.545	< 0.001	0.042	0.007	< 0.001	< 0.001
	c	236	235	236	234	236	151	231	236	236	234	117	140	178
GGT	a	−0.474^*^	−0.031	−0.064	−0.420^*^	−0.243^*^	1.000	−0.420^*^	0.453^*^	0.119	0.196^*^	−0.520^*^	0.571^*^	−0.464^*^
	b	< 0.001	0.710	0.432	< 0.001	0.003		< 0.001	< 0.001	0.146	0.016	< 0.001	< 0.001	< 0.001
	c	151	151	151	151	151	151	147	151	151	151	74	93	115
Bil	a	0.465^*^	0.349^*^	0.488^*^	0.746^*^	0.500^*^	−0.420^*^	1.000	−0.166^*^	−0.318^*^	−0.174^*^	0.369^*^	−0.535^*^	0.517^*^
	b	< 0.001	< 0.001	< 0.001	< 0.001	< 0.001	< 0.001		0.006	< 0.001	0.007	< 0.001	< 0.001	< 0.001
	c	275	231	232	229	231	147	275	272	274	243	126	145	185
Iron	a	−0.306^*^	−0.092	−0.048	−0.213^*^	0.040	0.453^*^	−0.166^*^	1.000	−0.083	0.040	−0.563^*^	0.381^*^	−0.323^*^
	b	< 0.001	0.160	0.462	0.001	0.545	< 0.001	0.006		0.166	0.534	< 0.001	< 0.001	< 0.001
	c	280	236	236	234	236	151	272	280	279	249	127	150	187
TP	a	−0.357^*^	−0.051	−0.221^*^	−0.269^*^	−0.263^*^	0.119	−0.318^*^	−0.083	1.000	0.791^*^	0.055	0.401^*^	−0.140
	b	< 0.001	0.427	0.001	< 0.001	< 0.001	0.146	< 0.001	0.166		< 0.001	0.533	< 0.001	0.051
	c	291	246	244	235	236	151	274	279	291	256	132	156	195
Glob	a	−0.220^*^	−0.033	−0.051	−0.165^*^	−0.133^*^	0.196^*^	−0.174^*^	0.040	0.791^*^	1.000	0.122	0.329^*^	−0.098
	b	< 0.001	0.612	0.430	0.012	0.042	0.016	0.007	0.534	< 0.001		0.161	< 0.001	0.173
	c	257	242	242	233	234	151	243	249	256	257	133	156	193
CRP	a	0.406^*^	0.149	0.248^*^	0.441^*^	0.248^*^	−0.520^*^	0.369^*^	−0.563^*^	0.055	0.122	1.000	−0.401^*^	0.444^*^
	b	< 0.001	0.100	0.006	< 0.001	0.007	< 0.001	< 0.001	< 0.001	0.533	0.161		< 0.001	< 0.001
	c	135	123	123	116	117	74	126	127	132	133	135	123	115
AB levels	a	−0.652^*^	−0.441^*^	−0.429^*^	−0.665^*^	−0.450^*^	0.571^*^	−0.535^*^	0.381^*^	0.401^*^	0.329^*^	−0.401^*^	1.000	−0.666^*^
	b	< 0.001	< 0.001	< 0.001	< 0.001	< 0.001	< 0.001	< 0.001	< 0.001	< 0.001	< 0.001	< 0.001		< 0.001
	c	166	147	146	139	140	93	145	150	156	156	123	166	132
Ii	a	0.348^*^	0.347^*^	0.340^*^	0.587^*^	0.322^*^	−0.464^*^	0.517^*^	−0.323^*^	−0.140	−0.098	0.444^*^	−0.666^*^	1.000
	b	< 0.001	< 0.001	< 0.001	< 0.001	< 0.001	< 0.001	< 0.001	< 0.001	0.051	0.173	< 0.001	< 0.001	
	c	222	188	184	178	178	115	185	187	195	193	115	132	222

Refer to additional file 2 for complete correlation analysis. (*HES* hematological score, *ALT* alanine transaminase, *ALP* alkaline phosphatase, *AST* aspartate transaminase, *CK* creatine kinase, *GGT* gamma-glutamyl transferase, *Bil* bilirubin, *TP* total protein, *Glob* globulin, *CRP* c-reactive protein, *AB* antibody, *Ii* infection intensity)

The HES showed a strong negative correlation with the antibody level (ρ = -0.652, *P* < 0.001), and a moderate positive correlation with the CRP level (ρ = 0.406, *P* < 0.001). Moderate to strong correlations were also found between levels of CRP and iron (ρ = −0.563), GGT (ρ = −0.520), bilirubin (ρ = 0.369), and platelet count (ρ = −0.359) (*P* < 0.001 each). Albumin showed moderate to strong positive correlations with total protein (ρ = 0.714), hematocrit (ρ = 0.586) and platelet counts (ρ = 0.376), and a moderate negative correlation with bilirubin (ρ = −0.303) (*P* < 0.001 each).

The *Babesia* spp. antibody levels showed strong negative correlations with the level of parasitemia (ρ = −0.666), the HES (ρ = − 0.652), bilirubin (ρ = −0.535), and AST (ρ = −0.665) (*P* < 0.001 each). Strong positive correlations were found between antibody levels and leukocyte count (ρ = 0.605) as well as platelet count (ρ = 0.571) (*P* < 0.001 each). There were moderate to strong correlations between the level of parasitemia and bilirubin (ρ = 0.517), CRP (ρ = 0.444), and the HES (ρ = 0.348) (*P* < 0.001 each).

## Discussion

Our study investigated acute-phase proteins, *Babesia* spp. antibody levels, and levels of parasitemia in dogs with acute *B. canis* infection, including the highest number of submitted blood samples in 342 dogs in Germany so far. Additionally, it was the first study taking the level of parasitemia determined by ddPCR into consideration and correlating the above-mentioned parameters with the number of parasites per milliliter EDTA blood. Our study demonstrated a moderate positive correlation between the determined level of parasitemia and the HES, indicating that a higher parasite burden leads to more significant hematologic abnormalities. Another interesting finding was that dogs with high antibody levels showed less severe clinicopathological alterations as well as lower levels of parasitemia in the peripheral blood.

In previous studies on acute *B. canis* infections, clinical manifestations were not correlated with the level of parasitemia [16, 29, 30], and no differences in parasitemia were detected between survivors and non-survivors by microscopy [19]. The parasitemia evaluated by microscopy was usually low with < 1% of erythrocytes infected with *B. canis* [31], ranging from 0.5 to 3.1% with a median of 1.2% in the most recent study [19]. Low parasitemia detected by microscopy was a common finding in dogs with severe clinical manifestations of *B. canis* infections [16, 29, 31]. However, to the best of our knowledge, neither Spearman’s nor Pearson’s correlation analysis was performed in previous studies.

The PCR methodology applied in our study detected the single copy *Bc28.1* gene; therefore, overestimation of the number of *B. canis* parasites seemed unlikely. PCR evaluates genetic material and cannot differentiate between living or dead organisms; however, this aspect seemed to be negligible due to the acute onset of disease in canine *B. canis* infections. In contrast to PCR, the microscopic evaluation of 5000 erythrocytes and more to estimate the level of parasitemia is time-consuming, and thus a challenge in routine diagnostics in clinical veterinary practice and commercial laboratories. Thus, PCR quantification could provide an economic and fast diagnostic alternative method. However, non-quantitative microscopy is an immediate and reliable diagnostic tool in emergency cases, especially in capillary smears providing a higher level of parasitemia of 12–100% compared with peripheral blood smears [17, 18]. Negative results in microscopy should be confirmed by PCR testing, as parasites might have been missed, especially in peripheral blood smears.

In addition to the observed impact of the level of parasitemia, individual *B. canis Bc28.1* genotypes were known to impact the severity of clinical signs and hematological as well as biochemical parameters [32]. In the past, three different genotypes were described circulating in Europe with regional impact. Genotype A was predominating in North-Eastern Europe, genotype B in South-Western Europe, and genotype 34 was identified in France, mostly as coinfection with genotypes A and/or B [32]. However, no information regarding the virulence of the mentioned genotypes was available [32]. In a recent study in Germany, various genotypes and the possibility of simultaneous infection with several genotypes were described [33]. The impact of different genotypes on hematological and biochemical results could not be ruled out in our study, as no genotyping was performed. Nevertheless, complicated *B. canis* infections with mortality rates up to 20% were most often observed in Central and Eastern Europe [8, 13, 34, 35], whereas in Western and Southern Europe predominantly uncomplicated infections with up to 5% mortality rate were reported [11, 14, 16, 36, 37]. The impact of *B. canis* genotypes on resulting levels of parasitemia, clinical as well as clinicopathological parameters, and outcome of infections needs to be further investigated.

Considering the country of origin for imported dogs, particularly high antibody levels were predominantly found in Eastern European countries (e.g., Romania, Hungary), whereas dogs from southern European countries (e.g., Greece, Croatia, Italy) tended to have lower antibody levels. These findings were linked to the above-mentioned regional differences in mortality rates in Europe [8, 11, 13, 14, 16, 34–37].

Exposure to *Babesia vogeli*, frequently occurring in southern Europe, leads to negative or low positive antibody levels, which was a possible explanation for low antibody levels in these areas [1, 25]. Also, serological cross-reactions with other protozoan parasites, predominantly *Leishmania* spp., were noted in *Babesia* spp. antibody testing [1, 25]. However, this was most likely not relevant in the present study, as only low numbers of dogs imported from Southern Europe were included.

As expected, the present study showed a strong negative correlation between the levels of parasitemia and antibodies. Additionally, severe hematological abnormalities represented by a high HES were associated with high levels of parasitemia, but low *Babesia* spp. antibody levels. Previous studies demonstrated that dogs vaccinated with a soluble parasite antigen (SPA) of *Babesia* species were protected against severe clinical manifestations of babesiosis [38–40]. Vaccinated dogs showed minor changes in blood parameters and no or less clinical manifestations [38–40]. Similar to antibody formation following vaccination, the development of protective antibodies against severe manifestations of disease after natural *B. canis* infection was considered likely in the present study. The latter is further supported by the fact that seropositive dogs with higher antibody levels had lower levels of parasitemia in their blood. In an experimental study, treatment with imidocarb dipropionate was effective in clearing the infection, but impaired the maintenance of protective antibodies, making dogs more susceptible for reinfection [41]. Pre-established immunity due to previous exposure to *B. canis* or vaccination was therefore very likely and refers to establishment of *Babesia* spp. antibody levels protecting against more severe clinical and clinicopathological abnormalities during following acute *B. canis* infections. Protective immunity developed from three weeks after booster vaccination onwards and remained effective for a period of at least another six months [42].

Additionally, the correlation analysis showed strong negative correlations between CRP and level of antibodies as well as strong positive correlations between CRP and the levels of parasitemia. CRP is a major positive acute-phase protein in dogs and therefore a marker for the severity of inflammatory reactions [43]. CRP is characterized by its very rapid increase (4–6 h after insult with a maximum after 24–48 h), which can be up to 50–100-fold [43]. An increase of > 100 μg/ml was described for *B. canis* infections [44, 45], which was confirmed in this study (Fig. 2). Therefore, an acute-phase response was expected in dogs with acute *B. canis* infection in the present study. It was also not surprising that the severity of CRP elevations was associated with the levels of parasitemia, which was also observed in other protozoal diseases as e.g., canine leishmaniasis, where the levels of parasitemia correlated with the severity of clinical as well as clinicopathological findings [46, 47].

Most commonly, dogs from Germany without stays abroad and traveled dogs showed low antibody levels against *Babesia* spp., suggesting that most of these dogs were not exposed to the pathogen in the recent past prior the acute onset of the *B. canis* infection. On the other hand, most dogs with import history showed high antibody levels, which indicates previous pathogen exposure and protection against severe hematological abnormalities and severe acute-phase reactions.

In the past, pigmenturia due to hemolytic anemia was considered a typical clinical/hematological finding for acute infections with *B. canis* in dogs [10, 48]. In contrast, this study showed (most often marked) thrombocytopenia as the most important hematologic abnormality, as it was already documented in recent studies [8, 9, 13, 14]. The cause of thrombocytopenia in acute babesiosis is not yet fully understood, but sequestering platelets in the spleen, destruction by macrophages, immune mediated thrombocytopenia induced by inflammatory cytokines, and NETosis were discussed as underlying causes [49, 50]. In dogs infected with *Babesia gibsoni*, anti-erythrocyte and anti-platelet antibodies were detected, suggesting an immune-mediated component in the development of anemia and thrombocytopenia [51]. A disseminated intravascular coagulation in severe cases of babesiosis might contribute to thrombocytopenia by platelet consumption [52, 53], but was only seen in about 20% of dogs with babesiosis [50]. Given the assumption that CRP correlated with the severity of disease, the negative correlation between platelet counts and CRP levels determined in the present study supported this hypothesis.

The biochemical results in this study were consistent with those described in previous studies [8, 13]. Hyperbilirubinemia, the most common abnormality, correlated positively with CRP and the level of parasitemia, and negatively with antibody levels. Hyperbilirubinemia is more likely to be associated with a more severe course of the disease. In the absence of profound changes in liver enzymes or liver function parameters in most dogs, hemolysis was a conceivable cause of hyperbilirubinemia in the present study due to acute *B. canis* infection, underlined by the strong positive correlation of levels of parasitemia with bilirubin levels.

Hypoalbuminemia would be expected in infected dogs as albumin is classified as a negative acute-phase protein [45, 54]. Earlier studies on *B. canis* infections in dogs have so far shown varying results, with some reporting low albumin levels and others reporting albumin in the reference range [8, 13, 45, 55, 56]. In the relatively large study population of the present study only a low percentage of dogs (10.5%) showed hypoalbuminemia. This may be explained by an individual disease severity of each dog, and, most likely, different time frames between onset of the infection and blood sampling. About every fourth dog showed elevated creatinine values (21.6%) and almost every second dog (45.2%) elevated urea, making prerenal azotemia in acute *B. canis* infection most likely.

Limitations of this study included the retrospective study design and the lack of biochemical, antibody, and level of parasitemia analyses in some dogs. Obvious limitations in questionnaires included the partial unavailability of complete datasets for all dogs and the number of different individual veterinarians involved. The questionnaires consisted of yes–no answers and free text entry was not possible. All dogs were additionally tested for *A. phagocytophilu*m, other co-infections were not excluded. A further limitation was the lack of *B. canis* genotyping. An influence of concomitant diseases on blood parameters could not be ruled out, as e.g., chronic vector-borne diseases as leishmaniosis and ehrlichiosis in dogs imported from the Mediterranean.

## Conclusions

Acute *B. canis* infection should be considered in dogs from Germany even without history of stays abroad, especially if presenting with severe thrombocytopenia. The levels of parasitemia determined by ddPCR were associated with hematological abnormalities, biochemical parameters, and acute-phase proteins in dogs with acute *B. canis* infection. Furthermore, dogs with high levels of parasitemia showed more severe clinicopathologic changes and low anti-*Babesia* antibody levels. Imported dogs showed significantly higher antibody levels compared with dogs with no history of stays abroad, indicating past exposure to the pathogen. These high antibody levels were associated with less severe clinicopathologic abnormalities, consistent with a protective nature of the antibodies and pre-established immunity. Further studies are needed to investigate the potential impact of different *B. canis* genotypes.

## Supplementary Information

Additional file 1. Text S1: Questionnaire sent to the referring veterinarian asking for medical history, potential stays abroad, clinical signs, therapy, and outcome in dogs with acute *Babesia canis* infection in Germany.

Additional file 2. Table S1: Correlation analysis (Spearman-Rho) showing correlations of laboratory findings in dogs with acute *Babesia canis* infection in Germany.

## Data Availability

Data supporting the main conclusions of this study are included in the manuscript.

## Abbreviations

AB: Antibodies
ALP: Alkaline phosphatase
ALT: Alanine transaminase
AST: Aspartate transaminase
CI: Confidence interval
CK: Creatine kinase
CRP: C-reactive protein
DNA: Deoxyribonucleic acid
EDTA: Ethylenediaminetetraacetic acid
ELISA: Enzyme-linked immunosorbent assay
GLDH: Glutamate dehydrogenase
GGT: Gamma-glutamyl transferase
HES: Hematological score
IFAT: Immuno-fluorescence-antibody-test
PCR: Polymerase chain reaction
PQ: Pathogen quantification
SD: Standard deviation
SIRS: Systemic inflammatory response syndrome
