# Supplementary material for: Impact of levels of parasitemia and antibodies, acute-phase proteins, as well as stays abroad on hematological and biochemical parameters in 342 dogs with acute Babesia canis infection
Source: Parasit Vectors. 2025 Aug 15;18:347. doi: 10.1186/s13071-025-06997-4 (PMC12355803; doi:10.1186/s13071-025-06997-4)
Supplement: Supplementary file 1 — Additional file 1. Text S1: Questionnaire sent to the referring veterinarian asking for medical history, potential stays abroad, clinical signs, therapy, and outcome in dogs with acute Babesia canis infection in Germany. [file 13071_2025_6997_MOESM1_ESM.pdf]

Labor für klinische Diagnostik GmbH & Co. KG

Laboklin GmbH & Co. KG
RESEARCH
Steubenstr. 4
97688 Bad Kissingen
Germany

Fragebogen

Bitte Praxisstempel und Kundennummer angeben

Offer-Nr.:
KC 24032

Study

Babesien

Besitzer: ______________________

Postleitzahl Besitzer: ______________________

Tierart und Name Hund ________________

Rasse: ______________________

Geschlecht: ______________________

Alter/Geburtsdatum: ______________________

Allgemeinuntersuchung Erstvorstellung

Allgemeinzustand: ____________________________

Rektale Körpertemperatur: ____________________________
(in °C)

Lymphknotenpalpation: ____________________________

Schleimhautfarbe/-feuchte: ____________________________

Kapilläre Füllungszeit: ____________________________

Zeckenbefall bei Vorstellung: O ja O nein

Fragen

1. Auslandsaufenthalte O ja O nein

   Falls ja: O Import O Reise

   Land: ______________________

   Zeitraum: ______________________

2. Zeckenbefall vorberichtlich O ja O nein

   Falls ja, wann _____________________

3. Ektoparasitenschutz bei Vorstellung O ja O nein

   Falls ja: Präparat: ________________________

   Zuletzt angewandt am: _________________

4. Klinik
   - Hämoglobinurie O ja O nein
   - Blutungen/Petechien O ja O nein

5. Outcome/Therapie
   - Verlauf: O unkompliziert O kompliziert
   - Carbesia Dosierung ____________ O ja O nein

6. Bekannte Grunderkrankungen O ja O nein

   Falls ja: Welche: _______________________

Die Daten werden entsprechend der Datenschutzgrundverordnung (DSGVO) anonymisiert verarbeitet. Die Umfrage ist freiwillig. Es werden keine Besitzernamen und -anschriften erhoben, auch werden keine IP-Adressen oder ähnliches ausgelesen. Die von uns erhobenen Daten werden nach den Richtlinien des Datenschutzgesetzes streng vertraulich behandelt. Eine Weitergabe an Dritte ist grundsätzlich ausgeschlossen. Die Tierdaten sind mittels einer ID-Nummer verschlüsselt, Daten des Besitzers werden mit Ausnahme der Postleitzahl nicht verarbeitet.
