# Supplementary material for: Impact of levels of parasitemia and antibodies, acute-phase proteins, as well as stays abroad on hematological and biochemical parameters in 342 dogs with acute Babesia canis infection
Source: Parasit Vectors. 2025 Aug 15;18:347. doi: 10.1186/s13071-025-06997-4 (PMC12355803; doi:10.1186/s13071-025-06997-4)
Supplement: Supplementary file 2 — Additional file 2. Table S1: Correlation analysis (Spearman-Rho) showing correlations of laboratory findings in dogs with acute Babesia canis infection in Germany. [file 13071_2025_6997_MOESM2_ESM.pdf]

Additional file 2: Table S1:

Correlation analysis (Spearman-Rho) showing correlations of laboratory findings in dogs with acute *Babesia canis* infections in Germany.

Asterisks mark statistically significant *P*-values < 0.05.

a = correlation coefficient, b = significance (two-sided), c = n

(HES: hematological score; ALT: alanine transaminase; ALP: alkaline phosphatase; AST: aspartate transaminase; CK: creatine kinase; GGT: gamma-glutamyl transferase; CRP: c-reactive protein; AB: antibodies; Ii: infection intensity)

		HES	ALT	ALP	AST	CK	GGT	GLDH	Lipase DGGR	Bilirubin	Urea	Creatinine	Triglyce-rides	Iron	Total protein	Globulin	Albumin	CRP	*B. canis* AB	Ii
HES	a	1.000	0.168*	0.343*	0.413*	0.265*	-0.474*	-0.055	0.183*	0.465*	0.205*	0.077	0.253*	-0.306*	-0.357*	-0.220*	-0.265*	0.406*	-0.652*	0.348*
	b		0.008	< 0.001	< 0.001	< 0.001	< 0.001	0.400	0.004	< 0.001	0.001	0.224	< 0.001	< 0.001	< 0.001	< 0.001	< 0.001	< 0.001	< 0.001	< 0.001
	c	342	249	244	235	236	151	238	249	275	250	250	207	280	291	257	257	135	166	222
ALT	a	0.168*	1.000	0.538*	0.651*	0.343*	-0.031	0.637*	0.143*	0.349*	0.278*	0.217*	0.114	-0.092	-0.051	-0.033	-0.024	0.149	-0.441*	0.347*
	b	0.008		< 0.001	< 0.001	< 0.001	0.710	< 0.001	0.024	< 0.001	< 0.001	0.001	0.102	0.160	0.427	0.612	0.710	0.100	< 0.001	< 0.001
	c	249	249	243	235	235	151	237	248	231	248	248	207	236	246	242	243	123	147	188
ALP	a	0.343*	0.538*	1.000	0.561*	0.344*	-0.064	0.415*	0.248*	0.488*	0.312*	0.172*	0.354*	-0.048	-0.221*	-0.051	-0.261*	0.248*	-0.429*	0.340*
	b	< 0.001	< 0.001		< 0.001	< 0.001	0.432	< 0.001	< 0.001	< 0.001	< 0.001	0.007	< 0.001	0.462	0.001	0.430	< 0.001	0.006	< 0.001	< 0.001
	c	244	243	244	234	236	151	233	244	232	244	244	206	236	244	242	243	123	146	184
AST	a	0.413*	0.651*	0.561*	1.000	0.699*	-0.420*	0.434*	0.382*	0.746*	0.568*	0.467*	0.359*	-0.213*	-0.269*	-0.165*	-0.220*	0.441*	-0.665*	0.587*
	b	< 0.001	< 0.001	< 0.001		< 0.001	< 0.001	< 0.001	< 0.001	< 0.001	< 0.001	< 0.001	< 0.001	0.001	< 0.001	0.012	0.001	< 0.001	< 0.001	< 0.001
	c	235	235	234	235	234	151	226	235	229	234	234	206	234	235	233	233	116	139	178
CK	a	0.265*	0.343*	0.344*	0.699*	1.000	-0.243*	0.276*	0.294*	0.500*	0.431*	0.266*	0.277*	0.040	-0.263*	-0.133*	-0.245*	0.248*	-0.450*	0.322*
	b	< 0.001	< 0.001	< 0.001	< 0.001		0.003	< 0.001	< 0.001	< 0.001	< 0.001	< 0.001	< 0.001	0.545	< 0.001	0.042	< 0.001	0.007	< 0.001	< 0.001
	c	236	235	236	234	236	151	228	236	231	236	236	205	236	236	234	235	117	140	178
GGT	a	-0.474*	-0.031	-0.064	-0.420*	-0.243*	1.000	0.227*	-0.140	-0.420*	-0.072	-0.163*	-0.022	0.453*	0.119	0.196*	-0.002	-0.520*	0.571*	-0.464*
	b	< 0.001	0.710	0.432	< 0.001	0.003		0.005	0.086	< 0.001	0.380	0.046	0.793	< 0.001	0.146	0.016	0.976	< 0.001	< 0.001	< 0.001
	c	151	151	151	151	151	151	149	151	147	151	151	151	151	151	151	151	74	93	115
GLDH	a	-0.055	0.637*	0.415*	0.434*	0.276*	0.227*	1.000	0.209*	0.152*	0.298*	0.167*	0.100	0.216*	0.013	-0.004	0.009	-0.133	-0.122	0.061
	b	0.400	< 0.001	< 0.001	< 0.001	< 0.001	0.005		0.001	0.022	< 0.001	0.010	0.155	0.001	0.847	0.953	0.896	0.149	0.147	0.414
	c	238	237	233	226	228	149	238	237	224	238	238	203	229	235	232	233	119	143	179
Lipase DGGR	a	0.183*	0.143*	0.248*	0.382*	0.294*	-0.140	0.209*	1.000	0.411*	0.388*	0.429*	0.309*	0.012	0.011	0.136*	-0.175*	0.274*	-0.233*	0.210*
	b	0.004	0.024	< 0.001	< 0.001	< 0.001	0.086	0.001		< 0.001	< 0.001	< 0.001	< 0.001	0.849	0.858	0.035	0.006	0.002	0.005	0.004
	c	249	248	244	235	236	151	237	249	232	248	248	207	236	247	242	243	123	147	188
Bilirubin total	a	0.465*	0.349*	0.488*	0.746*	0.500*	-0.420*	0.152*	0.411*	1.000	0.587*	0.452*	0.445*	-0.166*	-0.318*	-0.174*	-0.303*	0.369*	-0.535*	0.517*
	b	< 0.001	< 0.001	< 0.001	< 0.001	< 0.001	< 0.001	0.022	< 0.001		< 0.001	< 0.001	< 0.001	0.006	< 0.001	0.007	< 0.001	< 0.001	< 0.001	< 0.001
	c	275	231	232	229	231	147	224	232	275	232	232	202	272	274	243	243	126	145	185
Urea	a	0.205*	0.278*	0.312*	0.568*	0.431*	-0.072	0.298*	0.388*	0.587*	1.000	0.690*	0.532*	0.018	-0.149*	0.006	-0.269*	0.109	-0.251*	0.309*
	b	0.001	< 0.001	< 0.001	< 0.001	< 0.001	0.380	< 0.001	< 0.001	< 0.001		< 0.001	< 0.001	0.778	0.020	0.924	< 0.001	0.230	0.002	< 0.001
	c	250	248	244	234	236	151	238	248	232	250	250	206	237	246	243	244	124	148	189
Crea-tinine	a	0.077	0.217*	0.172*	0.467*	0.266*	-0.163*	0.167*	0.429*	0.452*	0.690*	1.000	0.304*	-0.191*	0.199*	0.258*	0.004	0.200*	-0.077	0.280*
	b	0.224	0.001	0.007	< 0.001	< 0.001	0.046	0.010	< 0.001	< 0.001	< 0.001		< 0.001	0.003	0.002	< 0.001	0.949	0.026	0.351	< 0.001
	c	250	248	244	234	236	151	238	248	232	250	250	206	237	246	243	244	124	148	189
Tri-glyce-rides	a	0.253*	0.114	0.354*	0.359*	0.277*	-0.022	0.100	0.309*	0.445*	0.532*	0.304*	1.000	0.018	-0.217*	-0.072	-0.261*	0.164	-0.254*	0.169*
	b	< 0.001	0.102	< 0.001	< 0.001	< 0.001	0.793	0.155	< 0.001	< 0.001	< 0.001	< 0.001		0.793	0.002	0.306	< 0.001	0.082	0.003	0.032
	c	207	207	206	206	205	151	203	207	202	206	206	207	205	207	206	206	113	134	161
Iron	a	-0.306*	-0.092	-0.048	-0.213*	0.040	0.453*	0.216*	0.012	-0.166*	0.018	-0.191*	0.018	1.000	-0.083	0.040	-0.220*	-0.563*	0.381*	-0.323*
	b	< 0.001	0.160	0.462	0.001	0.545	< 0.001	0.001	0.849	0.006	0.778	0.003	0.793		0.166	0.534	< 0.001	< 0.001	< 0.001	< 0.001
	c	280	236	236	234	236	151	229	236	272	237	237	205	280	279	249	249	127	150	187
Total protein	a	-0.357*	-0.051	-0.221*	-0.269*	-0.263*	0.119	0.013	0.011	-0.318*	-0.149*	0.199*	-0.217*	-0.083	1.000	0.791*	0.714*	0.055	0.401*	-0.140
	b	< 0.001	0.427	0.001	< 0.001	< 0.001	0.146	0.847	0.858	< 0.001	0.020	0.002	0.002	0.166		< 0.001	< 0.001	0.533	< 0.001	0.051
	c	291	246	244	235	236	151	235	247	274	246	246	207	279	291	256	256	132	156	195
Globulin	a	-0.220*	-0.033	-0.051	-0.165*	-0.133*	0.196*	-0.004	0.136*	-0.174*	0.006	0.258*	-0.072	0.040	0.791*	1.000	0.219*	0.122	0.329*	-0.098
	b	< 0.001	0.612	0.430	0.012	0.042	0.016	0.953	0.035	0.007	0.924	< 0.001	0.306	0.534	< 0.001		< 0.001	0.161	< 0.001	0.173
	c	257	242	242	233	234	151	232	242	243	243	243	206	249	256	257	256	133	156	193
Albumin	a	-0.265*	-0.024	-0.261*	-0.220*	-0.245*	-0.002	0.009	-0.175*	-0.303*	-0.269*	0.004	-0.261*	-0.220*	0.714*	0.219*	1.000	-0.020	0.297*	-0.083
	b	< 0.001	0.710	< 0.001	0.001	< 0.001	0.976	0.896	0.006	< 0.001	< 0.001	0.949	< 0.001	< 0.001	< 0.001	< 0.001		0.823	< 0.001	0.254
	c	257	243	243	233	235	151	233	243	243	244	244	206	249	256	256	257	133	156	193
CRP	a	0.406*	0.149	0.248*	0.441*	0.248*	-0.520*	-0.133	0.274*	0.369*	0.109	0.200*	0.164	-0.563*	0.055	0.122	-0.020	1.000	-0.401*	0.444*
	b	< 0.001	0.100	0.006	< 0.001	0.007	< 0.001	0.149	0.002	< 0.001	0.230	0.026	0.082	< 0.001	0.533	0.161	0.823		< 0.001	< 0.001
	c	135	123	123	116	117	74	119	123	126	124	124	113	127	132	133	133	135	123	115
B. canis AB	a	-0.652*	-0.441*	-0.429*	-0.665*	-0.450*	0.571*	-0.122	-0.233*	-0.535*	-0.251*	-0.077	-0.254*	0.381*	0.401*	0.329*	0.297*	-0.401*	1.000	-0.666*
	b	< 0.001	< 0.001	< 0.001	< 0.001	< 0.001	< 0.001	0.147	0.005	< 0.001	0.002	0.351	0.003	< 0.001	< 0.001	< 0.001	< 0.001	< 0.001		< 0.001
	c	166	147	146	139	140	93	143	147	145	148	148	134	150	156	156	156	123	166	132
Ii	a	0.348*	0.347*	0.340*	0.587*	0.322*	-0.464*	0.061	0.210*	0.517*	0.309*	0.280*	0.169*	-0.323*	-0.140	-0.098	-0.083	0.444*	-0.666*	1.000
	b	< 0.001	< 0.001	< 0.001	< 0.001	< 0.001	< 0.001	0.414	0.004	< 0.001	< 0.001	< 0.001	0.032	< 0.001	0.051	0.173	0.254	< 0.001	< 0.001	
	c	222	188	184	178	178	115	179	188	185	189	189	161	187	195	193	193	115	132	222
